# Leonurine alleviates lung ischemia–reperfusion injury through suppression of ferroptosis via RORα in male mice

**DOI:** 10.1530/JOE-25-0298

**Published:** 2026-01-19

**Authors:** Wanying Chen, Li Yang, Yincong Xue, Yuting Zhang, Chengshui Chen, Shuai Huang

**Affiliations:** ^1^ Department of Psychiatry, The First Affiliated Hospital of Wenzhou Medical University, Wenzhou, China; ^2^ Department of Pulmonary and Critical Care Medicine, The First Affiliated Hospital of Wenzhou Medical University, Wenzhou, China; ^3^ Department of Medicine and Therapeutics, The Chinese University of Hong Kong, Prince of Wales Hospital, Hong Kong SAR, China; ^4^ Zhejiang Province Engineering Research Center for Endoscope Instruments and Technology Development, Quzhou, China; ^5^ Department of Pulmonary and Critical Care Medicine, The Quzhou Affiliated Hospital of Wenzhou Medical University, Quzhou People’s Hospital, Quzhou, China; ^6^ Key Laboratory of Interventional Pulmonology of Zhejiang Province, The First Affiliated Hospital of Wenzhou Medical University, Wenzhou, China

**Keywords:** lung ischemia–reperfusion injury, leonurine, ferroptosis, RORα

## Abstract

Lung ischemia–reperfusion injury (LIRI) is a complex pathological condition that significantly impairs clinical outcomes following lung transplantation and thoracic surgery. Leonurine (LEO), an alkaloid derived from Leonurus japonicus, which has known anti-inflammatory and antioxidant properties, has shown therapeutic potential in various oxidative stress-related diseases. However, the effects of LEO on LIRI and its underlying mechanisms remain unclear. In the present study, a murine model of LIRI was established using wild-type mice. LEO treatment significantly improved lung histopathology, reduced oxidative stress, decreased pulmonary edema, and enhanced survival. Bioinformatics analyses – including volcano plot, KEGG enrichment, and GSEA – identified ferroptosis as a key regulatory pathway. *In vivo* and *in vitro* assays (HE, 4-HNE, and DHE labeling; immunofluorescence; and immunoblotting) confirmed that LEO inhibited ferroptosis in lung tissue and in MLE-12 cells. Mechanistically, LEO upregulated the RORα/Nrf2/GPX4 axis, thereby reducing lipid peroxidation and iron overload, as validated by BODIPY581/591 C11 and FeRhoNox-1 staining. Moreover, RORα inhibition abolished the anti-ferroptotic effects of LEO, indicating that its protective function is RORα dependent. Molecular docking further supported a potential direct interaction between LEO and RORα. Collectively, LEO alleviates LIRI by inhibiting ferroptosis through activation of the RORα/Nrf2/GPX4 signaling pathway. These findings suggest that LEO may serve as a promising therapeutic agent for the treatment of LIRI.

## Introduction

Lung ischemia–reperfusion injury (LIRI), commonly occurring after lung transplantation or thoracic trauma, arises when blood flow is reintroduced into an ischemic graft following implantation. This pathological event is a major contributor to primary graft dysfunction and is associated with high morbidity and mortality. LIRI frequently leads to aggravated pulmonary edema and acute respiratory distress syndrome, which significantly increase the risk of mortality, especially in patients requiring mechanical ventilation and those prone to pulmonary infections (1, 2). Despite its clinical relevance, the precise molecular mechanisms underlying LIRI remain poorly understood, and effective therapeutic strategies are still lacking.

Ferroptosis is a newly characterized, iron-dependent form of regulated cell death that is distinct from apoptosis, necrosis, and autophagy. It is driven by the accumulation of lethal lipid peroxides, facilitated by excess ferrous iron (Fe^2+^) and reactive oxygen species (3). Disruption of the system Xc^−^/glutathione (GSH)/glutathione peroxidase 4 (GPX4) axis – in particular, GSH depletion or GPX4 inactivation – has been identified as a core mechanism driving ferroptosis (3). Alveolar epithelial cell (AEC) ferroptosis has been implicated in various pathological conditions, including sepsis, acute liver injury (4), chronic obstructive pulmonary disease (COPD) (5), and lung transplantation-related complications (6, 7). Given that AECs are essential for maintaining pulmonary function and are among the first targets during lung injury (8), their susceptibility to ferroptosis may play a central role in the pathogenesis of LIRI. Although emerging evidence suggests that ferroptosis is associated with LIRI (9), the precise regulatory mechanisms remain unclear.

Leonurine (LEO), an alkaloid extracted from the traditional Chinese herb Leonurus japonicus, possesses multiple pharmacological properties, including anti-inflammatory, antioxidant, and endocrine-modulating effects (10). Recent studies have demonstrated LEO’s protective efficacy in models of respiratory disease, such as ovalbumin-induced asthma (11), highlighting its potential as a therapeutic agent in pulmonary pathologies. In addition, LEO has been reported to alleviate damage induced by lipopolysaccharide (LPS, 1 g/mL, 48 h) in the BEAS-2B human lung epithelial cell model of acute lung injury (12). However, these findings remain preliminary, with the underlying mechanisms largely limited to descriptions of oxidative stress and inflammation. Research on LEO’s role in regulating cell death pathways, including ferroptosis, particularly in animal models or through molecular interaction studies such as docking analyses, is still lacking. Therefore, further investigation is needed to comprehensively elucidate the mechanisms by which LEO affects LIRI.

The retinoic acid receptor-related orphan receptor alpha (RORα) is a nuclear transcription factor expressed in multiple tissues, including the brain, liver, immune system, and lungs (13). RORα plays a critical role in various physiological processes, including circadian rhythm regulation, lipid metabolism, immune response, and ferroptosis (14, 15). Dysregulation of RORα has been associated with metabolic syndromes, neurodegenerative disorders, and pulmonary diseases. Meanwhile, the nuclear factor erythroid 2-related factor 2 (Nrf2) is a key transcriptional regulator involved in antioxidant defense and cellular stress responses. Increasing evidence suggests that the Nrf2 pathway plays an essential role in ferroptosis, particularly in the context of lung diseases (16). Nevertheless, the potential interaction and regulatory axis among LEO, RORα, and Nrf2 in LIRI-associated ferroptosis remains undefined and warrants further investigation.

## Materials and methods

### Animals

Male C57BL/6 mice were obtained from the Laboratory Animal Center of the First Affiliated Hospital of Wenzhou Medical University (Wenzhou, China). RORα-knockout (KO) and wild-type (WT) C57BL6 mice were purchased from Jackson Laboratory. All animal procedures complied with the Animal Management Rule of the PRC Ministry of Health and the NIH Guide for the Care and Use of Laboratory Animals and were approved by the Animal Care Committee of Wenzhou Medical University.

### *In vivo* LIRI model

The LIRI model was established as described previously (17). Mice were anesthetized with sodium pentobarbital (80 mg/kg i.p.), and body temperature (37°C) was maintained. Mechanical ventilation was applied using an RSP1002 ventilator with an I:E ratio of 1:1, a tidal volume of 10 mL/kg, and 100% FiO_2_. Buprenorphine (0.1 mg/kg s.c.) was administered before a left thoracotomy. The left pulmonary hilum was occluded for 1 h, followed by 2 h of reperfusion, as described in Ref. (18).

### Cell culture and reagent

Mouse lung epithelial (MLE)-12 cells were maintained in DMEM. For the hypoxia/reoxygenation (HR) model, cells were serum-starved for 12 h and then incubated under 95% N_2_ and 5% CO_2_ for 8 h, followed by reoxygenation in normoxic conditions for 12 h (18). For genetic manipulation, RORα-shRNA (CAG​TCG​GGA​TTG​GAC​ATC​AAT) and sh-scramble lentiviruses were obtained from Genomeditech. Deferoxamine (Defer) was from AbMole (M5558); LEO (HY-N0741) and Erastin (HY-15763) were from MedChemExpress. The corresponding mice were injected intraperitoneally with above reagents (30 mg/kg LEO for 6 days (19), 40 mg/kg Erastin for 3 days (20), and 100 mg/kg Defer for 5 days (21)).

### Lung wet-to-dry ratios

The lung wet-to-dry weight (W/D) ratio was calculated to evaluate the degree of pulmonary edema. First, the lung tissue was isolated from the mice and measured in its wet state after washing with saline before being dried in an oven at 80°C for 12 h and reweighed; then, the lung wet-to-dry weight ratio was calculated.

### Dihydroethidium (DHE) labeling

Lung tissues were embedded in OCT, frozen in liquid nitrogen, cut into 8 μm sections, stored at −80°C, stained with 25 μM DHE at 37°C for 30 min, washed, and imaged under fluorescence microscopy.

### Analysis of the protein concentration in bronchoalveolar lavage fluid (BALF)

Lungs were lavaged with saline via tracheal cannulation. BALF protein concentration was measured using a Bradford Protein Quantification Kit (P0006 C, Beyotime Institute of Biotechnology, China) following the manufacturer’s instructions.

### Lung myeloperoxidase (MPO) activity assay

Lung tissues were homogenized in cold isotonic saline and centrifuged at 3,000 *g* for 15 min. The MPO activity was measured using a commercial kit (Nanjing Jiancheng Bioengineering Institute, Nanjing, China) according to the manufacturer’s instructions.

### 4-Hydroxynonenal staining (4-HNE)

4-HNE was used to measure the level of lipid peroxidation. First, the isolated lung tissues were fixed in 4% paraformaldehyde, embedded in paraffin, cut into 5 μm slices, and immunostained for 4-HNE following the standard protocol. The slices were then incubated with 3% hydrogen peroxide, blocked with goat serum, and incubated with mouse anti-4-hydroxynonenal antibody (1:100, MAB3249-SP, R&D Systems, USA) overnight. After that, the slices were incubated with horseradish peroxidase-conjugated goat anti-mouse IgG (1:500, #31430, Fisher Scientific, USA) for 1 h. Finally, color was developed using a diaminobenzoate solution (Cat. Number: DA1010, Solarbio, China), and the slices were counterstained with hematoxylin.

### Lactate dehydrogenase (LDH) assay

LDH is a classic marker of tissue or cell injury (22) and was evaluated using an LDH assay kit (A020-2, Nanjing Jiancheng Biotechnology, China) as previously described (23). In brief, blood samples were collected from the facial vein using a 4 mm needle; after 5–9 drops of blood were obtained, pressure was applied to stop the bleeding with hemostatic cotton. Blood was allowed to clot at room temperature for 30–60 min and then centrifuged at 4,688 ***g*** for 10–15 min at 4°C. The clear serum was transferred to a sterile tube. The serum was incubated with kit reagents. After incubation for 5 min, absorbance was measured at 450 nm according to the manufacturer’s instructions (24).

### BODIPY 581/591 C11 staining

BODIPY 581/591 C11 reagent (Invitrogen, USA, D3861, 10 μM) was added to cells and incubated for 30 min at 37 °C. Cells were washed three times with PBS, and fluorescence was read at separate wavelengths: excitation/emission at 581/591 nm for the reduced dye and excitation/emission at 488/510 nm for the oxidized dye. The ratio of the emitted fluorescence intensity at 591 to that at 510 nm gives a readout of lipid peroxidation in the cells.

### FeRhoNox-1 staining

FeRhoNox-1 (MKBio, USA, MK4558) staining was used to evaluate the Fe^2+^ concentration at the cellular level. In brief, FeRhoNox-1 was dissolved in DMSO and then diluted to 5 μM with PBS. The cells were incubated with 5 μM FeRhoNox-1 at 37°C for 30 min and then washed three times with PBS solution, and images were observed under a fluorescence microscope (wavelengths of emission at 570 nm).

### Bioinformatics analysis

Gene Expression Omnibus (GEO) raw data were obtained from the GEO database (GSE127003), which were deposited by Wong A, Zamel R, Yeung J, Bader GD, Dos Santos CC, Bai X, Wang Y, Keshavjee S, and Liu M. The GSE127003 data were generated from human lung transplantation, lung samples originated from 46 patients at the end of cold ischemia and after 2 h of reperfusion following transplantation. Detailed information is shown at https://www.ncbi.nlm.nih.gov/geo/query/acc.cgi?acc=GSE127003. GSE127003 was analyzed using the DESeq R package (1.36.0) based on the negative binomial distribution generalized linear model and the Benjamini–Hochberg method. Differentially expressed genes (DEGs) were identified with |log2(fold change)|≥1, *P*-value <0.05. Pathway enrichment analysis was performed using the clusterProfiler R package based on the Kyoto Encyclopedia of Genes and Genomes (KEGG) database (https://www.genome.jp/kegg/). The *P*-value was calculated by a hypergeometric test. Gene set enrichment analysis (GSEA) was performed using the R package ClusterProfiler based on the significance threshold set at a false discovery rate of <0.25 and *P* < 0.05.

### Molecular docking and molecular dynamics (MD) simulation

The three-dimensional (3D) structure of LEO was retrieved from the PubChem database (https://pubchem.ncbi.nlm.nih.gov/). The amino acid sequence of retinoic acid receptor-related orphan receptor alpha (RORα) was obtained from the UniProt database. To predict the tertiary structure of RORα, AlphaFold3 was employed, followed by energy minimization using the Rosetta Relax protocol to improve structural reliability. The resulting protein structure was further processed using PyMOL for structural preparation, including removal of water molecules and addition of hydrogen atoms.

Blind molecular docking was performed using the DeepMice online server (https://www.deepmice.com/), which applies artificial neural networks for cavity recognition and ligand–receptor docking. The docking poses were ranked based on the docking score provided by DeepMice, and the top-ranked complex was selected for subsequent interaction analysis and MD simulations to assess binding stability.

MD simulations were conducted using GROMACS 2023.3 with a total simulation time of 100 ns. Prior to production runs, energy minimization was carried out using the steepest descent algorithm within a canonical (NVT) ensemble. The minimization parameters included a step size of 0.01 nm and a maximum force threshold of 1,000 kJ/mol nm. After energy minimization, the system was equilibrated through a 100 ps NVT simulation at constant volume, during which the temperature was gradually increased from 0 to 310.15 K to allow uniform solvent distribution. This was followed by a 100 ps NPT equilibration phase using the Berendsen barostat to stabilize the system pressure at 1 bar.

During the MD simulation, all bonds involving hydrogen atoms were constrained using the LINCS algorithm. Electrostatic interactions were treated using the particle-mesh Ewald (PME) method with a cutoff distance of 1.2 nm. Non-bonded interactions were calculated with a cutoff of 10 Å and updated every 10 steps. The integration time step was set to 2 fs. Periodic boundary conditions were applied throughout the simulation.

Trajectory analyses included calculations of root mean square deviation (RMSD), root mean square fluctuation (RMSF), radius of gyration (Rg), the number of hydrogen bonds, binding free energy (BFE), and construction of the free energy landscape (FEL) to evaluate the dynamic stability and interaction profile of the LEO–RORα complex.

### Immunofluorescence staining

Cells were treated with MitoTracker CMXRos (200 nM) (Invitrogen) and incubated with anti-prostaglandin-endoperoxide synthase 2 (PTGS2) and secondary antibodies, as described in our previous studies. For dual immunofluorescence staining, the cells were incubated with the primary antibody at 4°C overnight, followed by incubation with the secondary antibody (Proteintech, USA) at 37°C for 1 h. DAPI was used for nuclear staining. Immunofluorescence images were obtained using an inverted fluorescence microscope. Colocalization data were quantified using the ImageJ software.

### Immunoblotting analysis

Proteins were extracted from cells or tissues and subjected to sodium dodecyl sulfate-polyacrylamide gel electrophoresis (SDS-PAGE) on Tris–glycine gels, as described previously (25). The engaged primary antibodies include GPX4 (52455, Cell Signaling and Technology (CST), USA), PTGS2 (12282, CST), 4-HNE (AB48506, Abcam, UK), Nrf2 (12721, CST), and RORα (34639, CST), and the expression of β-actin (50201, Kemei Borui, China) was used as a loading control. All primary antibodies were used at a 1:1,000 dilution.

### Cell viability assay

Cell viability was quantitatively analyzed using a 3-(4,5-dimethyl-2-thiazolyl)-2,5-diphenyl-2H-tetrazolium bromide (MTT) assay (475989, Sigma-Aldrich, USA). In brief, experiments were conducted in 96-well plates, and the absorbance of each well was determined at 570 nm.

### RNA extraction and qRT-PCR

Total RNA was isolated from MLE-12 cells using Trizol reagent (BioTeke, China, RP1001) and reverse-transcribed into cDNA with SuperScript III (Vazyme, China, R223-01). Quantitative real-time PCR was conducted using reagents from Thermo Fisher Scientific (A31667) on a QuantStudio™ 3 real-time PCR system. β-actin was employed as the internal control, and relative mRNA expression levels were determined by the 2^−^^ΔΔCt^ method. Primer sequences are provided in Supplementary Table 1 (see section on Supplementary materials given at the end of the article).

### Statistical analysis

All data are depicted as mean ± SEM and analyzed using GraphPad Prism. Two-group comparisons were made by Student’s *t*-test, while multi-group comparisons were performed via one-way ANOVA followed by Tukey’s post hoc test, except experiments performed *in vivo*, which used two-way ANOVA. *P* < 0.05 was considered significant.

## Results

### Effect of LEO on LIRI in mice

**Figure 1:**
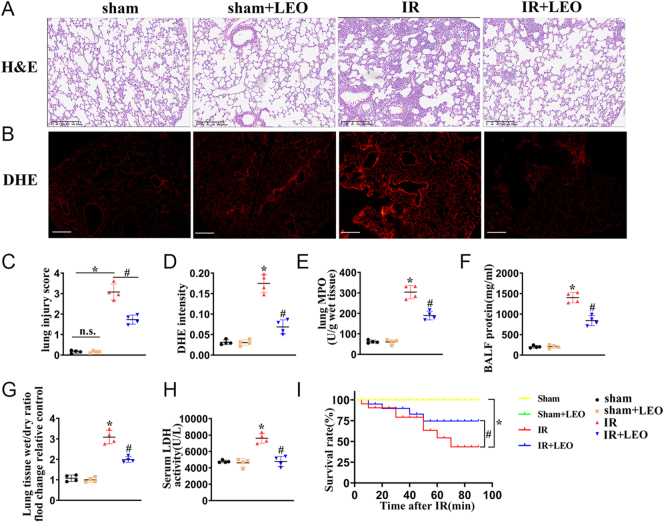
Effect of LEO on LIRI in mice. (A) Representative H&E-stained images from murine lung tissues after lung ischemia–reperfusion injury (IR) and IR + LEO. Scale bar: 200 μm. (B) Fluorescent images of superoxide levels in murine lung tissues. Superoxide was determined with the fluorescent indicator DHE, and the fluorescent intensity of DHE was observed with a computer-assisted microscope. Scale bar: 200 μm. (C) The lung pathological damage score illustrating the extent of lung injury in different groups. (D) Quantification of the DHE staining in murine lung tissue from (C). (E) The lung MPO concentrations were measured by an assay kit and quantified. (F) The total BALF protein was detected in lung tissue. (G) The ratio of dry lung weight to wet lung weight was analyzed. (H) Quantification of LDH release. (I) The survival rate was quantified in mice. The data are represented as mean ± SEM (*n* = 4). Significance: *P < 0.05 vs sham in mice, ^#^*P* < 0.05 vs IR in mice. A full color version of this figure is available at https://doi.org/10.1530/JOE-25-0298.

To evaluate the protective effect of LEO on LIRI, we first established a stable and reproducible mouse model. The experimental group was pretreated with LEO via gavage at a dose of 30 mg/kg/day for three consecutive days prior to LIRI induction. This pretreatment aimed to assess the potential benefits of LEO administration on LIRI. The lung wet-to-dry (W/D) weight ratio and the total protein concentration in bronchoalveolar lavage fluid (BALF) are commonly used indicators of pulmonary vascular permeability and lung injury severity. As shown in Fig. 1, LEO pretreatment significantly ameliorated various pathological changes in LIRI mice, including lung histopathology (Fig. 1A and C), superoxide accumulation (assessed by DHE staining, Fig. 1B and D), vascular permeability (BALF protein content, Fig. 1F), W/D ratio (Fig. 1G), neutrophil infiltration (MPO activity, Fig. 1E), overall tissue damage (LDH release, Fig. 1H), and survival rate (Fig. 1I). Taken together, these findings indicate that LEO effectively alleviates LIRI in mice.

### LIRI is associated with ferroptosis, and inhibition of ferroptosis alleviates lung injury in mice

**Figure 2:**
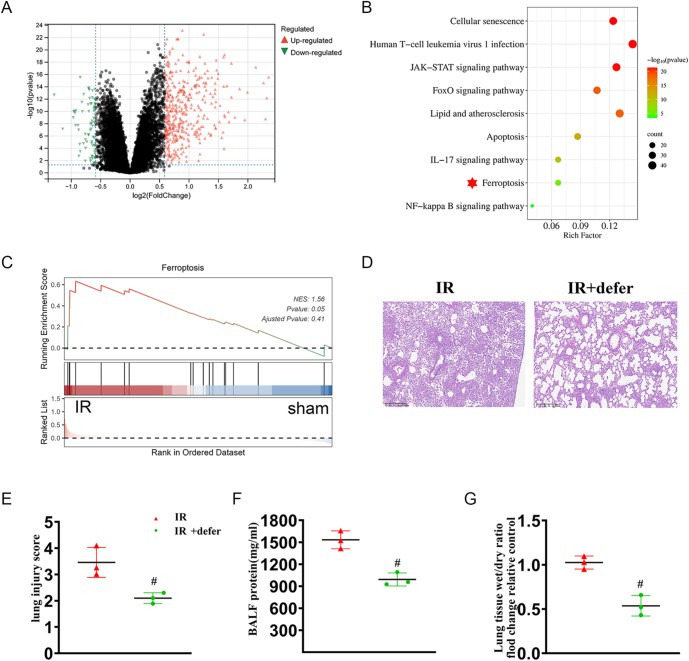
LIRI is associated with ferroptosis, and inhibition of ferroptosis alleviates lung injury in mice. (A) The volcano plot visually displays the distribution of differential genes from GEO database. (B) Gene ontology (GO) analysis between the sham group and IR group. (C) GSEA showing the significant enrichment of ferroptosis signaling in lung tissue between IR and sham groups. (D) Representative H&E-stained images from murine lung tissues after lung ischemia–reperfusion injury (IR) and IR + Defer. Scale bar: 200 μm. (E) Lung pathological damage score illustrating the extent of lung injury in different groups. (F) The total BALF protein was detected in lung tissue. (G) The ratio of dry lung weight to wet lung weight was analyzed. The data are represented as mean ± SEM (*n* = 3). Significance: ^#^*P* < 0.05 vs IR in mice. A full color version of this figure is available at https://doi.org/10.1530/JOE-25-0298.

To explore the molecular changes associated with LIRI, we conducted relevant bioinformatics analysis using the GEO database (GSE127003). The 2,601 genes of RNA-Seq data were shown (Fig. 2A). A total of 111 DEGs were identified, including 109 upregulated and 2 downregulated genes. Subsequent network pharmacology analysis revealed that the ferroptosis pathway was significantly enriched among these DEGs, as shown by the KEGG pathway analysis (Fig. 2B). Gene set enrichment analysis (GSEA) further confirmed the upregulation of ferroptosis in LIRI samples (Fig. 2C). To verify the involvement of ferroptosis in LIRI (9), we administered the ferroptosis inhibitor deferoxamine (Defer) *in vivo*. The results demonstrated that inhibition of ferroptosis significantly protected lung tissue against LIRI-induced damage (Fig. 2D, E, F). These findings suggest that ferroptosis plays a critical role in LIRI and that blocking this process provides therapeutic benefit.

### LEO alleviates the LIRI via ferroptosis inhibition

**Figure 3:**
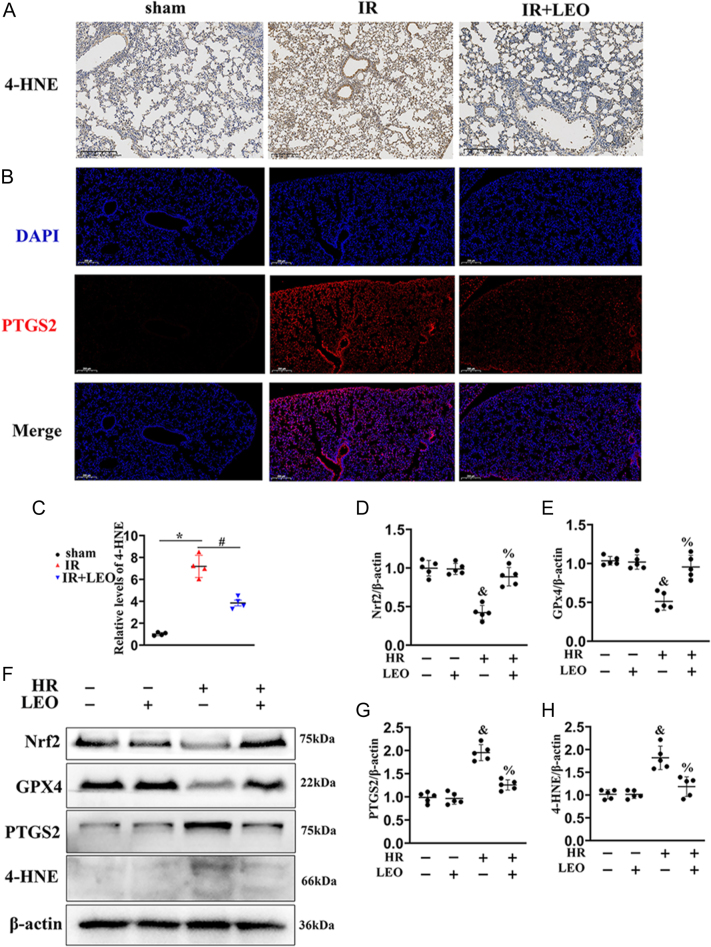
LEO alleviates the LIRI via ferroptosis inhibition. (A) Representative 4-HNE staining image in the lung of sham, IR, and IR + LEO. The positive staining (brown) demonstrated positive expression (*n* = 4). Scale bar = 200 μm. (B) Representative immunofluorescence of PTGS2 and DAPI in the lung of sham, IR, and IR + LEO. Scale bar = 200 μm. (C) Quantification of 4-HNE from (A). (D) Representative western blot and the quantitative analysis of (E) Nrf2/β-actin, (F) GPX4/β-actin, (G) PTGS2/β-actin, and (H) 4-HNE/β-actin in MLE-12 cells under different groups. Significance: *P < 0.05 vs sham, ^#^*P* < 0.05 vs IR, ^&^*P* < 0.05 vs con, ^%^*P* < 0.05 vs HR in MLE-12 cells. The immunoblotting data are represented as mean ± SEM (*n* = 5). A full color version of this figure is available at https://doi.org/10.1530/JOE-25-0298.

**Figure 4:**
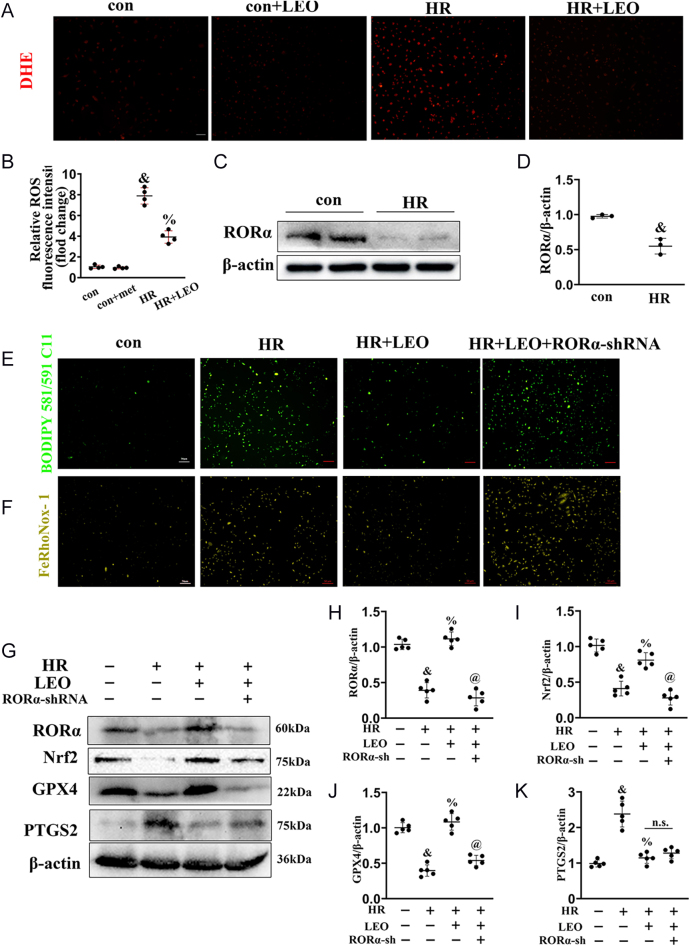
RORα/Nrf2/GPX4 signaling mediates the anti-ferroptotic effect of LEO in MLE-12 cells. (A) Representative DHE staining from MLE-12 cells. Scale bar: 100 μm. (B) The quantitative analysis of (A). (C) Cell lysates of MLE-12 cells were used to detect RORα protein levels by immunoblotting. (D) Quantitative analysis of (C). The data are represented as mean ± SEM (*n* = 3). (E and F) Representative pictures of immunofluorescence staining with the C11-BODIPY and FeRhoNox-1 probes for MLE-12 cells. Scale bar: 50 μm. (G) Representative western blot and the quantitative analysis of (H) RORα/β-actin, (I) Nrf2/β-actin, (J) GPX4/β-actin, and (K) PTGS2/β-actin in MLE-12 cells under different groups. Significance: ^&^*P* < 0.05 vs con. ^%^*P* < 0.05 vs HR. ^@^*P* < 0.05 vs HR + LEO in MLE-12 cells. The data are represented as mean ± SEM (*n* = 5). A full color version of this figure is available at https://doi.org/10.1530/JOE-25-0298.

LIRI is characterized by inflammation and regulated cell death, including ferroptosis (26). Considering the central role of ferroptosis in LIRI, we next examined whether the protective effect of LEO is mediated via ferroptosis suppression. Immunofluorescence staining for ferroptosis markers 4-HNE and PTGS2 showed a significant reduction in their expression following LEO treatment in LIRI mice (Fig. 3A, B, C), while no significance between sham and control groups was observed (Supplementary Fig. 2A and B). To further investigate the mechanism *in vitro*, MLE-12 cells were subjected to hypoxia–reoxygenation (HR) conditions (5% CO_2_, 1% O_2_, and 94% N_2_ at 37°C for 6 h, followed by 2 h of reoxygenation in 95% air and 5% CO_2_). The optimal concentration of LEO (400 μM for 24 h) was validated using the MTT assay (Supplementary Fig. 1A). Under HR conditions, LEO significantly improved cell viability (Supplementary Fig. 1B). Western blot analysis showed that LEO reduced the expression of ferroptosis markers 4-HNE and PTGS2 (Fig. 4D, G, H), while restoring the expression of ferroptosis antagonists GPX4 and Nrf2 (Fig. 4D, E, F). Collectively, these data confirm that LEO alleviates LIRI by inhibiting ferroptosis in both *in vivo* and *in vitro* models.

### RORα/Nrf2/GPX4 signaling mediates the anti-ferroptotic effect of LEO in MLE-12 cells

To investigate the mechanism by which LEO suppresses ferroptosis, DHE staining was performed. The results indicated that LEO pretreatment markedly reduced HR-induced oxidative stress and cytotoxicity (Fig. 4A and B). Given the emerging role of the transcription factor RORα in ferroptosis and airway inflammation (27), we assessed its expression. Both RORα mRNA and protein levels were significantly decreased under HR conditions compared to control (Fig. 4C and D, Supplementary Fig. 2C) but increased under HR + LEO conditions compared to HR (Fig. 4G and H, Supplementary Fig. 2C). Consequently, we speculated that LEO regulates the RORα at both the protein and transcriptional level. Meanwhile, we verified the efficiency of RORα shRNA for the next assay (Supplementary Fig. 1C). Furthermore, lipid peroxidation and Fe^2+^ accumulation – hallmarks of ferroptosis – were reduced by RORα knockdown using shRNA (Fig. 4E and F), supporting the role of RORα in mediating ferroptosis.

The immunoblotting was used to verify the RORα-related downstream ferroptosis signaling pathway. Immunoblotting showed that HR reduced the expression of RORα, Nrf2, and GPX4, while increasing 4-HNE and PTGS2 expression. These effects were reversed by LEO treatment (Fig. 4G and H), suggesting that LEO inhibits ferroptosis by activating the RORα/Nrf2/GPX4 signaling axis.

### The protective effect of LEO is RORα dependent in mice

**Figure 5:**
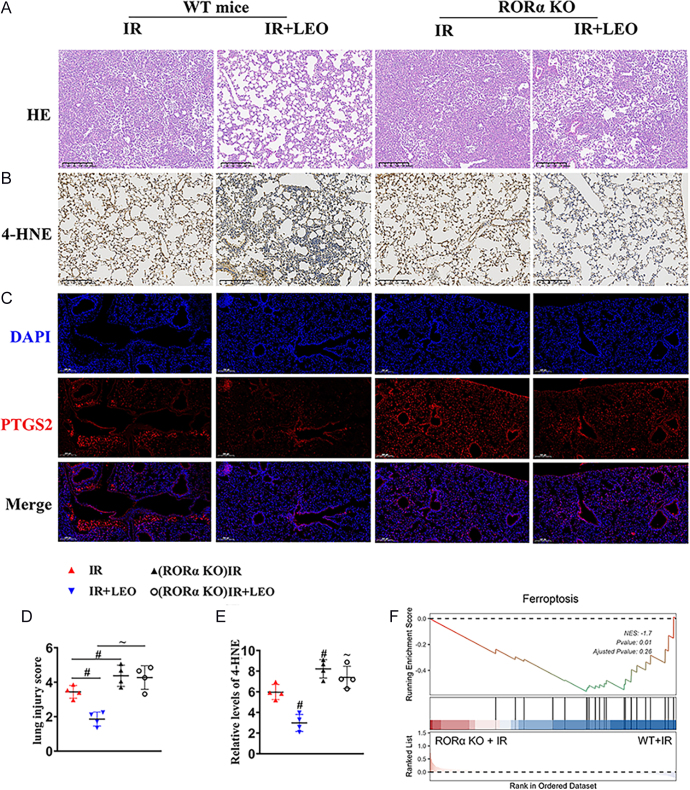
The protective effect of LEO is RORα dependent in mice. (A) Representative H&E staining from WT mice or RORα-KO mice in the presence of IR or IR + LEO. Scale bar: 200 μm. (B) Representative 4-HNE staining in lung tissue. Scale bar: 200 μm. (C) Representative immunofluorescence with DAPI and PTGS2 of lung tissue. Scale bar: 200 μm. (D and E) The extent of lung from (A) and (B) was quantified. (F) GSEA showing the significant enrichment of ferroptosis signaling in lung tissue between RORα-KO + IR and WT + IR. The data are represented as mean ± SEM (*n* = 4). Significance: ^#^*P* < 0.05 vs normal mice with IR; ∼ *P* < 0.05 vs normal mice with IR + LEO. All the above results in graphs from western blot were normalized to the first group. A full color version of this figure is available at https://doi.org/10.1530/JOE-25-0298.

To further elucidate the role of RORα, we employed RORα-knockout (RORα-KO) mice. Histological analysis showed that RORα-KO mice subjected to LIRI displayed severe lung injury that was not ameliorated by LEO administration (Fig. 5A and D). Consistently, ferroptosis markers 4-HNE and PTGS2 remained elevated in RORα-KO mice despite LEO treatment (Fig. 5B, C, E). Moreover, GSEA (Fig. 5F) confirmed that RORα deficiency significantly downregulated ferroptosis compared to WT mice under ischemia–reperfusion conditions. These results suggest that the protective effect of LEO against LIRI is mediated through RORα activation.

### Molecular docking reveals the potential interaction between LEO and RORα

**Figure 6:**
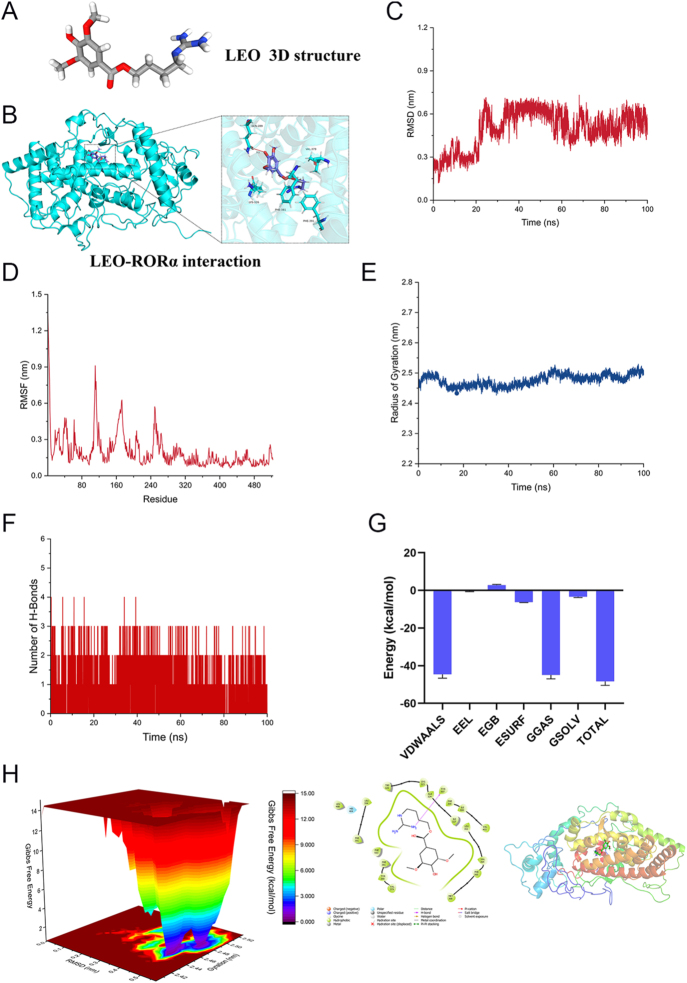
Molecular docking reveals the potential interaction between LEO and RORα. (A) The 3D structure of LEO obtained from the PubChem database. (B) Representative docking complex of RORα and LEO; docking score = 6.019. (C and D) Root mean square deviation (RMSD) and root mean square fluctuation (RMSF) values of the RORα–LEO docking complex. (E) The radius of gyration (Rg) characterizes the change in the looseness of the protein peptide chain during the simulation process. (F) The number of hydrogen bonds was used to simulate the change of the number of hydrogen bonds formed between small molecules and proteins with the simulation time. (G) The BFE analysis shows that the binding energy between small molecules and proteins is mainly van der Waals energy (−44.57 kcal/mol), and the electrostatic energy is −0.36 kcal/mol, indicating that electrostatic interaction is slightly conducive to the binding of proteins and small molecules, and the main interaction between small molecules and proteins is through van der Waals interaction. (H) The FEL analysis optimized the small molecule–protein interaction mode, and the small molecule and the protein maintained certain hydrophobic interactions and hydrogen bond interactions, further explaining the stability of the interaction between the small molecule and the protein. A full color version of this figure is available at https://doi.org/10.1530/JOE-25-0298.

LEO, as a small molecule compound, affects RORα expression according to our previous confirmation, and we explore the possibility of a direct interaction between LEO and RORα. Molecular docking technology was used to analyze interactions between LEO and its target proteins. The 3D structure of LEO was obtained from PubChem (Fig. 6A), and the RORα structure was retrieved from the AlphaFold Protein Structure Database (Fig. 6B). The docking score of 6.019 suggests a favorable interaction between LEO and RORα. MD simulations indicated that the LEO–RORα complex reached stability after 10 ns, as demonstrated by RMSD values (Fig. 6C). The low RMSF values and stable radius of gyration (Rg) further support the complex’s structural integrity (Fig. 6D and E). The number of hydrogen bonds remained stable during the simulation, reinforcing the strength of the interaction (Fig. 6F). FEL and BFE analyses corroborated the stability of the complex (Fig. 6G and H). These findings collectively suggest that LEO may directly bind to RORα, contributing to its ferroptosis-modulating effects.

## Discussion

Lung ischemia–reperfusion injury (LIRI) is a complex pathological process that poses a significant clinical challenge, particularly in the contexts of lung transplantation, cardiopulmonary bypass, and pulmonary embolism (28). It is characterized by oxidative stress, inflammatory responses, and various forms of regulated cell death. Among these, ferroptosis – a recently discovered iron-dependent form of programmed cell death marked by lipid peroxidation – has emerged as a key mechanism in the progression of LIRI (29, 30). Our study demonstrates that LEO, an alkaloid derived from Leonurus japonicus, significantly ameliorates LIRI by inhibiting ferroptosis through the activation of the RORα/Nrf2/GPX4 signaling pathway. We first established a reliable mouse model of LIRI and observed that LEO pretreatment significantly attenuated lung injury, as evidenced by reduced histopathological damage, vascular permeability, neutrophil infiltration, and oxidative stress. These protective effects were corroborated by improved survival rates and decreased levels of pro-injured cytokines, such as MPO and LDH. *In vitro*, LEO enhanced the viability of MLE-12 cells under hypoxia–reoxygenation conditions, confirming its cytoprotective effects. These findings are consistent with previous reports highlighting the anti-inflammatory and antioxidant properties of LEO in various tissue injury models (19, 31).

Bioinformatic analysis and gene expression profiling identified the signaling of senescence, JAK-Stat, apoptosis, and ferroptosis were enriched; although we selected the ferroptosis signaling pathway as a significantly enriched pathway in LIRI, the other pathways are worth further exploration. The ferroptosis inhibitor deferoxamine markedly reduced lung damage, underscoring the contribution of ferroptosis to LIRI pathogenesis. Notably, LEO treatment suppressed the expression of ferroptosis markers 4-HNE and PTGS2 while upregulating protective regulators such as GPX4 and Nrf2, both *in vivo* and *in vitro*. These observations suggest that LEO mitigates LIRI, at least in part, by inhibiting ferroptosis.

Nuclear factor erythroid 2-related factor 2 (Nrf2) is a master regulator of cellular antioxidant defense mechanisms. It promotes the expression of several cytoprotective genes, including GPX4 and SLC7A11, which are essential for lipid peroxide detoxification and maintenance of redox homeostasis (32). Our results show that LEO significantly enhances Nrf2 and GPX4 expression, supporting the hypothesis that LEO activates Nrf2-mediated antioxidant signaling to inhibit ferroptosis in lung epithelial cells.

A novel aspect of our study is the identification of retinoic acid receptor-related orphan receptor alpha (RORα) as a key upstream regulator of the Nrf2/GPX4 axis in LIRI. RORα has been implicated in lipid metabolism, circadian rhythm, and inflammation, and emerging evidence suggests its involvement in ferroptosis regulation (33, 34). We found that LIRI significantly reduced RORα expression, while LEO reversed this downregulation. Furthermore, RORα knockdown abolished the protective effects of LEO, leading to persistent ferroptotic damage and lung injury. This was further supported by experiments using RORα-knockout mice, in which LEO failed to exert protective effects, highlighting the indispensability of RORα in LEO-mediated cytoprotection.

Molecular docking and dynamics simulations further revealed a favorable binding affinity between LEO and RORα, indicating a potential direct interaction. The stability of the LEO–RORα complex, supported by RMSD, RMSF, Rg, hydrogen bond analyses, and free energy calculations, suggests that LEO may function as a natural agonist or modulator of RORα activity. This finding not only elucidates the mechanistic basis of LEO action but also provides a foundation for the development of novel RORα-targeted therapies in lung injury and ferroptosis-related diseases.

All animal experiments in the present study were performed using male mice. Male animals were selected to reduce biological variability associated with the estrous cycle, thereby improving experimental consistency and statistical power for mechanistic analyses. Accordingly, the relevance of these findings to females remains to be determined, and the underlying mechanisms in female mice require further investigation.

Despite these promising findings, several limitations should be acknowledged. First, while our *in vivo* and *in vitro* models provided robust evidence of the anti-ferroptotic effects of LEO, the precise molecular interface between LEO and RORα warrants further investigation through crystallography or nuclear magnetic resonance studies. Second, although RORα appears central to the protective effect of LEO, its interaction with other signaling pathways and transcription factors involved in ferroptosis remains to be fully elucidated. Finally, clinical validation in human samples or patient-derived organoids is necessary to translate these findings into therapeutic strategies.

## Conclusion

**Figure 7:**
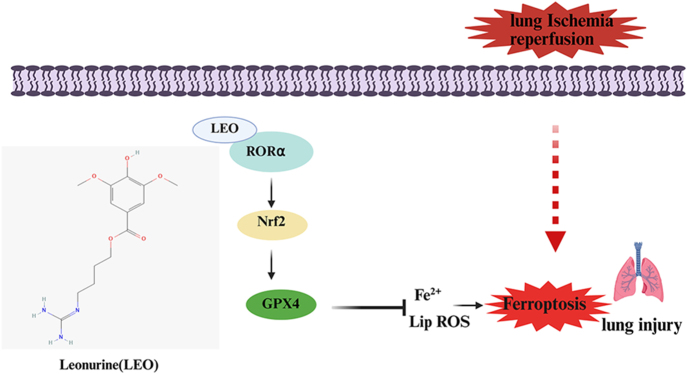
A mechanism map of leonurine on lung ischemia–reperfusion injury by inhibiting ferroptosis through RORα/Nrf2/GPX4 signaling. A full color version of this figure is available at https://doi.org/10.1530/JOE-25-0298.

Our study demonstrates for the first time that LEO confers protection against LIRI by inhibiting ferroptosis via the activation of the RORα/Nrf2/GPX4 signaling pathway (Fig. 7). These findings offer new insights into the molecular mechanisms underlying LIRI and suggest that LEO may serve as a promising candidate for the prevention and treatment of lung ischemia–reperfusion injury.

## Supplementary materials



## Declaration of interest

The authors declare that there is no conflict of interest that could be perceived as prejudicing the impartiality of the study reported.

## Funding

This study was supported by the Key Laboratory of Interventional Pulmonology of Zhejiang Province (2019E10014) and the National Natural Science Foundation of Chinahttps://doi.org/10.13039/501100001809 (82170017 and 82370085).

## Author contribution statement

SH and WYC designed the study. WYC conducted the main experiments and analyzed the data. SH analyzed the data and wrote the manuscript. WYC supervised the research and revised the manuscript. LY, YCX, and YTZ were responsible for the method. CSC provided all financial support. All authors read and approved the final manuscript.

## Data availability

The data from this study is available from the corresponding authors on reasonable request.
