# Supplementary material for: Leonurine alleviates lung ischemia–reperfusion injury through suppression of ferroptosis via RORα in male mice
Source: J Endocrinol. 2026 Jan 19;268(1):e250298. doi: 10.1530/JOE-25-0298 (PMC12910573; doi:10.1530/JOE-25-0298)
Supplement: Supplementary file 1 [file supplementary_materials.pdf]

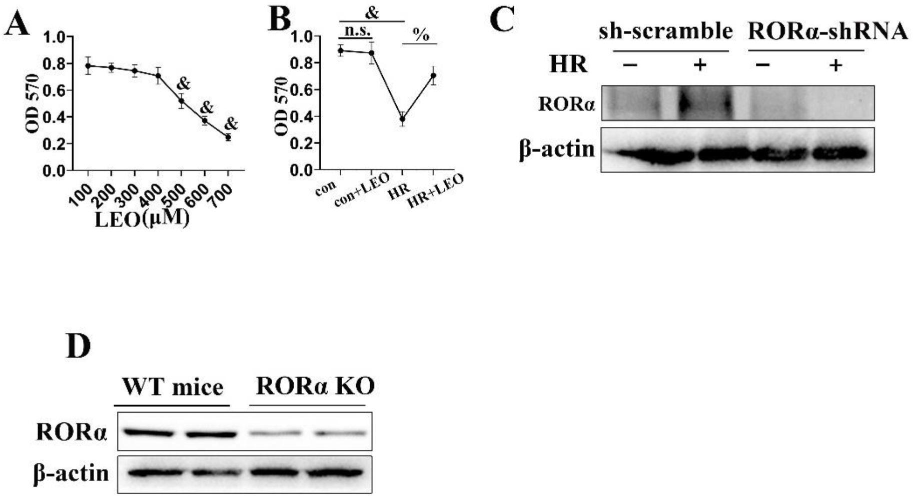

Supplementary figure 1. The effect of different LEO treatment on MLE-12 cells and verification of RORα silence in MLE-12 cells and RORα knockdown in mice. (A) MLE-12 cells viability was quantified in different concentration of LEO by MTT assay. (B) Quantification of MLE-12 cells viability. (C) and (D) Representative Western blot of RORα/β-actin in MLE-12 cells or lung tissue under different treatment. Significance: &P< 0.05 vs. con. %P < 0.05 vs.HR. The data are represented as the means±SEM (n=3).

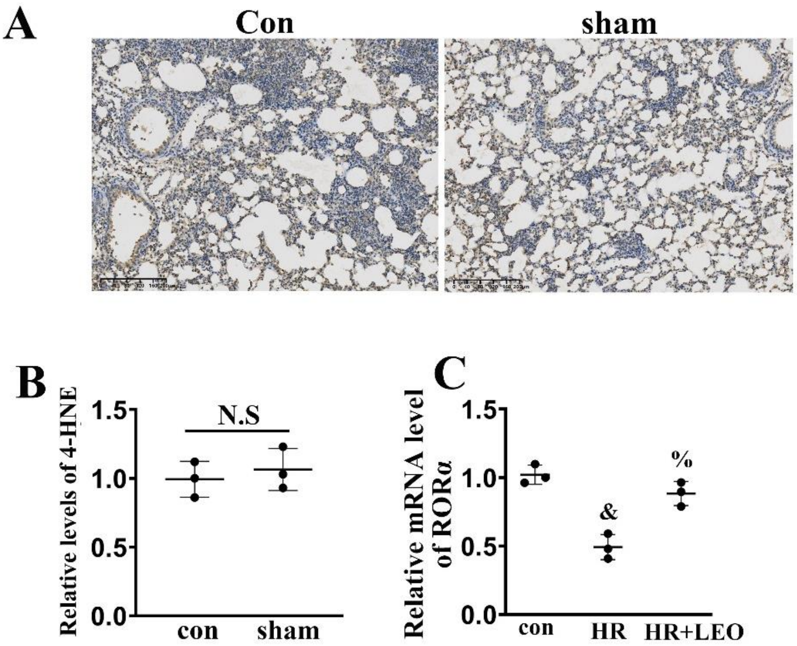

Supplementary figure 2. The 4-HNE staining in vivo and RORα mRNA level in vitro(MLE-12 cells). (A) Representative 4-HNE staining image in the lung of above group. The positive staining (brown) demonstrated positive expression (n = 3). Scale bars = 200μm. (B) The quantitative analysis of (A), (C) The quantitative analysis of RORα mRNA level. Significance: N.S means to no significance, &P< 0.05 vs. con. %P < 0.05 vs.HR. The data are represented as the means±SEM (n=3).

Supplementary Table 1. Primers used in quantitative PCR.

gene	sequence
RORα	5’-ATTCAGCTGGCCCTTCAGCAC-3’<br>5’-TGGAGGAAAATGGAGTCGCACAATG-3’
β-actin	5’-TTGTCAGCAATGCATCCTGCAC-3’<br>5’-GAAGGCCATGCCAGTGAGCTTC-3’
